# Early ^18^F-FDG uptake as a reliable imaging biomarker of T790M-mediated resistance but not MET amplification in non-small cell lung cancer treated with EGFR tyrosine kinase inhibitors

**DOI:** 10.1186/s13550-016-0229-0

**Published:** 2016-10-10

**Authors:** Viviana De Rosa, Francesca Iommelli, Marcello Monti, Ciro Gabriele Mainolfi, Rosa Fonti, Silvana Del Vecchio

**Affiliations:** 1 Institute of Biostructures and Bioimaging, National Research Council, Via T. De Amicis 95, 80145 Naples, Italy; 2 Department of Advanced Biomedical Sciences, University of Naples Federico II, Via Pansini 5, 80131 Naples, Italy

**Keywords:** ^18^F-FDG PET/CT, EGFR, Tyrosine kinase inhibitors, Resistance, MET amplification

## Abstract

**Background:**

The two main mechanisms of resistance to EGFR tyrosine kinase inhibitors (TKIs) in non-small cell lung cancer (NSCLC) are the occurrence of T790M secondary mutation in the kinase domain of EGFR and MET amplification. The aim of the present study was to test whether early changes of ^18^F-fluorodeoxyglucose (^18^F-FDG) uptake in animal models bearing erlotinib-resistant NSCLC may have different imaging patterns of response to erlotinib depending on the molecular mechanisms underlying resistance.

Animal tumor models were developed using NSCLC H1975 cells bearing the T790M mutation and H1993 cells with MET amplification. Nude mice bearing erlotinib-resistant H1975 and H1993 xenografts (four animals for each cell line and for each treatment) were subjected to ^18^F-FDG PET/CT scan before and immediately after treatment (50 mg/kg p.o. for 3 days) with erlotinib, WZ4002, crizotinib, or vehicle. A three-dimensional region of interest analysis was performed to determine the percent change of ^18^F-FDG uptake in response to treatment. At the end of the imaging studies, tumors were removed and analyzed for glycolytic and mitochondrial proteins as well as levels of cyclin D1.

**Results:**

Imaging studies with ^18^F-FDG PET/CT in H1975 tumor-bearing mice showed a reduction of ^18^F-FDG uptake of 25.87 % ± 8.93 % after treatment with WZ4002 whereas an increase of ^18^F-FDG uptake up to 23.51 % ± 9.72 % was observed after treatment with erlotinib or vehicle. Conversely, H1993 tumors showed a reduction of ^18^F-FDG uptake after treatment with both crizotinib (14.70 % ± 1.30 %) and erlotinib (18.40 % ± 9.19 %) and an increase of tracer uptake in vehicle-treated (56.65 % ± 5.65 %) animals. The in vivo reduction of ^18^F-FDG uptake was always associated with downregulation of HKII and p-PKM2 Tyr105 glycolytic proteins and upregulation of mitochondrial complexes (subunits I–IV) in excised tumors.

**Conclusions:**

^18^F-FDG uptake is a reliable imaging biomarker of T790M-mediated resistance and its reversal in NSCLC whereas it may not be accurate in the detection of MET-mediated resistance.

## Background

Non-small cell lung cancer (NSCLC) is the leading cause of cancer-related deaths in the world, showing only limited responsiveness to conventional anticancer agents. The development of molecularly targeted agents and in particular of tyrosine kinase inhibitors (TKIs) of the epidermal growth factor receptor (EGFR) provided alternative strategies for treating this disease [1]. Currently, advanced NSCLC patients are candidates to first-line therapy with EGFR TKIs such as gefitinib and erlotinib if their tumors bear activating mutations of EGFR that have been recognized as the major determinant of effective tumor response to these targeted agents [2]. A high rate of response to EGFR TKIs was indeed found in patients with advanced EGFR-mutant NSCLC resulting in an improvement of progression-free (PFS) and overall (OS) survival of these patients [3]. The clinical efficacy of gefitinib or erlotinib is however ultimately limited by the development of acquired resistance to EGFR TKIs in virtually all NSCLC patients with activating mutations of EGFR who initially respond to therapy [4, 5]. About half of resistant tumors develop T790M secondary mutations in EGFR which prevent an effective inhibition by EGFR TKIs [6, 7] whereas an additional 15–20 % of tumors undergoes amplification of MET receptor tyrosine kinase [8] which causes a lateral activation of EGFR signaling cascade despite receptor inhibition.

^18^F-Fluorodeoxyglucose (^18^F-FDG) and positron emission tomography/computed tomography (PET/CT) has been successfully employed in the diagnosis, staging, and treatment monitoring in NSCLC patients [9, 10]. The rationale to use ^18^F-FDG in the detection and staging of NSCLC resides in the fact that this tumor, as many other malignancies, has an oncogene-driven glycolytic phenotype that implies a high glucose demand due to an inefficient energy production and the consequent dependence of cancer cells from blood glucose supply. The rationale to use ^18^F-FDG in the assessment of tumor response to therapy relies on the fact that conventional cytotoxic agents, by inducing cell death, cause a reduction of cell viability and glucose demand with a consequent decrease of ^18^F-FDG uptake that may precede tumor shrinkage as assessed by morphovolumetric criteria. Previous studies in NSCLC patients also showed an early (2–14 days) reduction of ^18^F-FDG uptake in response to treatment with EGFR TKIs [11–14] that was predictive of morphovolumetric tumor response, PFS, and OS. In sensitive NSCLC cells, the rapid reduction of ^18^F-FDG uptake in response to EGFR TKIs was mainly correlated with the inhibition of the PI3K/AKT pathway, reduction of hexokinase activity, and downregulation of glucose transporters [15] that largely preceded any measurable changes in the percentage of cells undergoing apoptosis. In our previous studies, we showed that the inhibition of EGFR signaling in NSCLC cells inhibits aerobic glycolysis and restores oxidative phosphorylation through the concerted downregulation of hexokinase II (HKII) and pyruvate kinase M2 phosphorylated at Tyr105 (p-PKM2 Tyr105) and upregulation of mitochondrial complexes (OXPHOS) [16]. In particular, the selective inhibition of the AKT pathway in NSCLC cells resulted in the upregulation of OXPHOS mediated by peroxisome proliferator-activated receptor-γ coactivator-1α (PGC-1α) and increased levels of ATP whereas the parallel downregulation of the ERK1/2 pathway reduced the glycolytic cascade and lactate levels. Notably, NSCLC cells resistant to first-generation EGFR TKIs showed a differential metabolic response to erlotinib depending on the specific mechanism causing such resistance. On the basis of these observations, here, we tested whether early changes of the ^18^F-FDG uptake in animal models bearing erlotinib-resistant NSCLC may have different imaging patterns of response to erlotinib depending on the molecular mechanism underlying resistance. Furthermore, we tested whether imaging findings may reflect tumor levels of selected markers of energy metabolism.

## Methods

### Animal tumor models and treatment

**Fig. 1:**
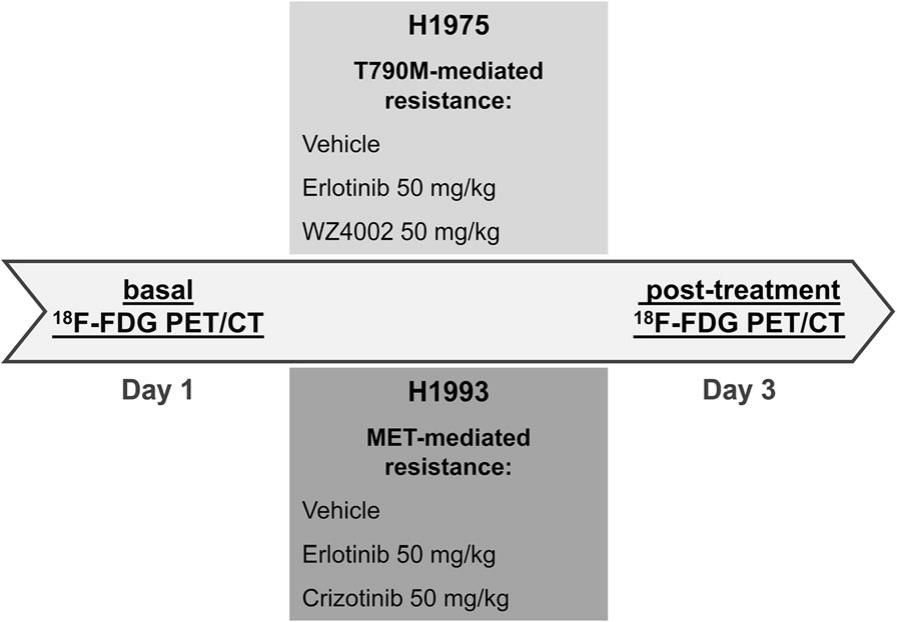
Representative scheme of treatment in H1975- and H1993-tumor-bearing animals. NSCLC animal models of T790M-mediated resistance (H1975) were treated by oral gavage with 50 mg/kg erlotinib, WZ4002, or vehicle whereas mice bearing xenografts with MET amplification (H1993) were treated with 50 mg/kg erlotinib, crizotinib, or vehicle. Treatment was started 3 h after the baseline ^18^F-FDG PET/CT scan on day 1 and was stopped 3 h before post-treatment scan on day 3

All animal experimental procedures were conducted in accordance with Italian law for animal protection and were approved by the Italian Ministry of Health-Animal Welfare Direction (Protocol No. DGSAF21940-A-16/11/2013). Female BALB/c (nu/nu) mice, 6 weeks old, weighing 15–20 g were purchased from Charles River Laboratories (Milan, Italy). Two NSCLC cell lines were obtained from the American Type Culture Collection. In particular, H1975 cells bear an activating point mutation in exon 21 (L858R) and also harbor the T790M mutation in the kinase domain of EGFR [17]. Despite the T790M-mediated resistance, H1975 cells remain EGFR-driven. H1993 cells are reported to have a high level of *MET* gene amplification (15 copy numbers) [18, 19] and wild-type EGFR, thus showing resistance to erlotinib [8]. All cells were grown in Roswell Park Memorial Institute (RPMI) medium supplemented with 10 % fetal bovine serum, 100 IU/mL penicillin, and 50 μg/mL streptomycin in a humidified incubator with 5 % CO_2_ at 37 °C, and then, 5–10 × 10^6^ cells were resuspended in 200 μl RPMI medium and injected s.c. into the flank of nude mice. When tumors reached a mean volume of approximately 100 mm^3^, animals were randomized into treatment groups (four animals for each cell line and for each treatment) and subjected to imaging studies. Tumor-bearing animals were treated daily for 3 days by oral gavage with 50 mg/kg of erlotinib, WZ4002 [20, 21] (an irreversible EGFR TKI with a higher affinity for T790M mutant EGFR than for wild-type EGFR), crizotinib [22, 23] (a MET inhibitor), or vehicle as described in Fig. 1.

Two additional subgroups of H1975- and H1993-tumor-bearing animals underwent longitudinal studies and were treated with 100 mg/kg erlotinib, WZ4002, crizotinib, or vehicle for 9 days. Tumor size was measured daily by caliper, and volume was determined using the following formula: volume = 0.5 × greatest diameter × (shortest diameter)^2^.

### Imaging studies with ^18^F-FDG and small-animal PET/CT

Each animal underwent a baseline and a post-treatment scan using a small-animal PET/CT scanner (eXplore Vista Pre-Clinical PET Scanner GE Healthcare). After fasting for 8 h, animals received 7.4 MBq of ^18^F-FDG by i.v. injection through the tail vein. Animals were anesthetized using 2 % isoflurane and then subjected to PET/CT scan at 60 min post-injection.

Body temperature of the animals was held constant during tracer biodistribution and imaging studies by heating pad or heat lamp. One bed position including the tumor was scanned, and CT images were acquired with the x-ray source set at 35 kVp and 200 μA for 10 min followed by PET image acquisition for 20 min. After acquisition, the images were reconstructed by a combined algorithm based on Fourier rebinning (FORE) followed by 2D iterative image reconstruction using ordered-subset expectation maximization (OSEM). The reconstructed images had a matrix size of 175 × 175 and a voxel size of 0.3875 × 0.3875 × 0.7750 mm^3^. PET images were corrected for decay and converted to SUV. No statistically significant change of animal weight was observed after treatment. PET/CT data were transferred in DICOM format to an OsiriX workstation (Pixmeo, Switzerland). Three-dimensional regions of interest were drawn around the tumor on transaxial PET images of the baseline and post-treatment scans, and a volume of interest was determined using an automated isocontouring program [23, 24]. The maximum SUV (SUV_max_) within the tumor volume of interest was then registered for each study. Finally, the percentage change of the ^18^F-FDG uptake in the post-treatment scan relative to baseline scan was determined for each animal. All quantitative data from animal imaging studies were expressed as mean ± SE.

### Analysis of excised tumors

After treatment, tumors were surgically removed, immediately frozen in liquid nitrogen, and stored at −80 °C until used. Tumor samples (at least three for each animal model and each treatment) were homogenized on ice in RIPA lysis buffer with protease and phosphatase inhibitors (Sigma-Aldrich) using a dounce homogenizer followed by passages through a 26-gauge needle. The suspension was clarified by centrifugation at 13,000×*g* for 30 min at 4 °C and subjected to western blot analysis using a standard procedure.

Since in a previous study [16] we showed that inhibition of EGFR signaling in NSCLC cells reduces aerobic glycolysis and restores oxidative phosphorylation through the concerted downregulation of HKII and p-PKM2 Tyr105 and upregulation of mitochondrial complexes (OXPHOS), we tested the levels of these selected markers of energy metabolism in tumors from untreated and treated animals. Antibodies used for western blotting included mouse monoclonal antibodies against actin (Sigma; 1 μg/mL) and α-tubulin (Sigma; 1 μg/mL), and the OXPHOS cocktail (Mitoscience, Eugene, OR; 1:1000) that targets the following proteins: 20-kD subunit of Complex I (20 kD), COX II of Complex IV (22 kD), 30-kD Ip subunit of Complex II (30 kD), core 2 of Complex III (~50 kD), and F1α (ATP synthase) of Complex V (~60 kD); rabbit monoclonal antibody against PKM2 (Cell Signaling; 0.1 mg/mL); and rabbit polyclonal antibodies against hexokinase II (Cell Signaling; 1:1000), phospho-PKM2 Tyr105 (Cell Signaling; 1:1000), cyclin D1 (Cell Signaling; 1:1000), and PGC-1α (Santa Cruz Biotechnology, 1:1000). A commercially available ECL kit (GE Healthcare, UK) was used to reveal the reaction.

The western blotting signal was then quantified by morphodensitometric analysis using ImageJ software (NIH, Bethesda, MD, USA). Briefly, the product of the area and the optical density of each band were determined and normalized to the same parameter derived from the actin control. Data were expressed as relative protein levels of each treated sample compared to the corresponding vehicle-treated internal control.

### Statistical analysis

Statistical analysis was done using the software MedCalc for Windows, version 12.7.0.0 (MedCalc Software, Mariakerke, Belgium). Unpaired or paired Student’s *t* test was used when appropriate to compare means. In particular, a paired *t* test was used to compare ^18^F-FDG uptake in the same tumors before and after treatment, whereas an unpaired *t* test was used to examine differences between untreated controls and treated groups. Differences between means were considered statistically significant for *p* < 0.05 (*) and highly statistically significant for *p* < 0.01 (**).

## Results

**Fig. 2:**
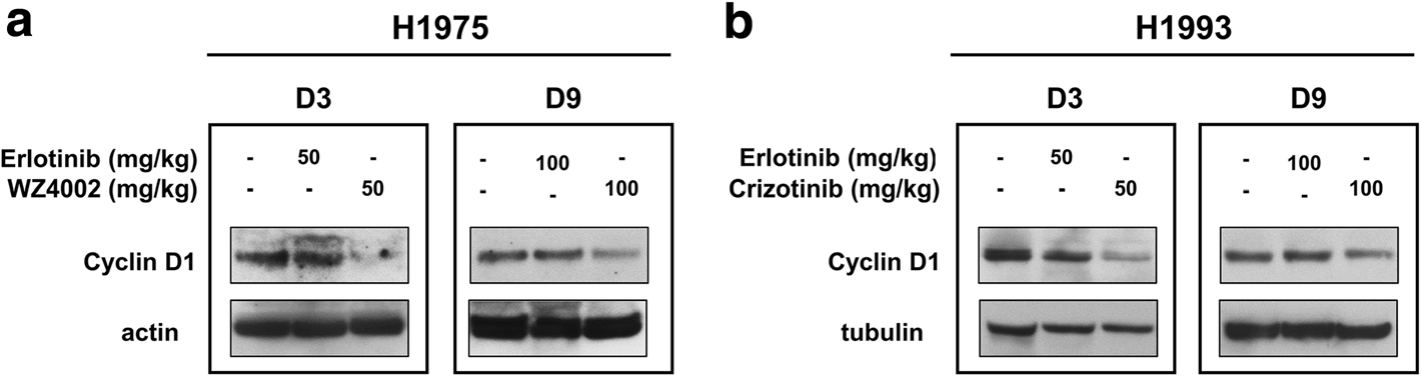
Cyclin D1 levels in xenografts obtained from untreated and treated animals. **a** H1975- and **b** H1993-tumor-bearing animals were treated for 3 or 9 days with 50 or 100 mg/kg erlotinib, WZ4002, crizotinib, or vehicle. Surgically removed tumors were homogenized and tested for levels of cyclin D1, one of the main terminal mediators of EGFR signaling pathways, by western blot analysis at the indicated time and doses. Actin or tubulin served to ensure equal loading

**Fig. 3:**
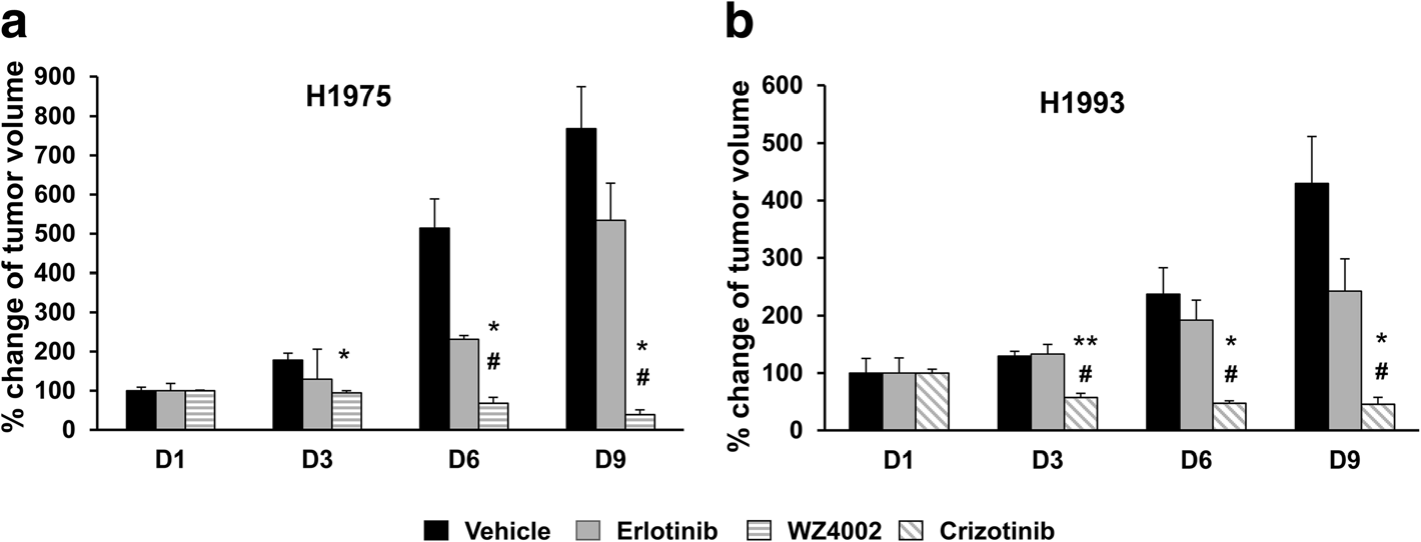
Long-term tumor growth of NSCLC xenografts in untreated and treated animals. Tumor growth of **a** H1975 and **b** H1993 xenografts in animals treated with 100 mg/kg of erlotinib, WZ4002, crizotinib, or vehicle. Data are expressed as a percentage of tumor volume at day 1 considered as 100 %. At each time point, significant differences versus vehicle-treated animals were indicated with *p < 0.05 and **p < 0.01 whereas the symbol ^#^ indicated significant differences versus erlotinib-treated animals, *p* < 0.05

To test whether H1975 and H1993 tumors were resistant to erlotinib and to demonstrate that animal treatment with WZ4002 or crizotinib could overcome T790M-mediated resistance or MET amplification, respectively, we determined the levels of cyclin D1, one of the terminal mediators of EGFR signaling pathways, in untreated and treated tumor-bearing animals (Fig. 2). Both short- (3 days) and long-term (9 days) treatment with erlotinib did not cause any significant change of cyclin D1 levels indicating resistance of H1975 (Fig. 2a) and H1993 (Fig. 2b) tumors to first-generation EGFR TKIs. Conversely, a strong reduction of cyclin D1 levels was observed after treatment of animals bearing H1975 or H1993 tumors with WZ4002 or crizotinib, respectively. Since short-term treatment with 50 mg/kg inhibitors did not cause any significant changes in tumor volume (data not shown), long-term studies were performed with high-dose treatment to confirm that H1975 (Fig. 3a) and H1993 (Fig. 3b) tumors were resistant to erlotinib and sensitive to WZ4002 or crizotinib, respectively.

**Fig. 4:**
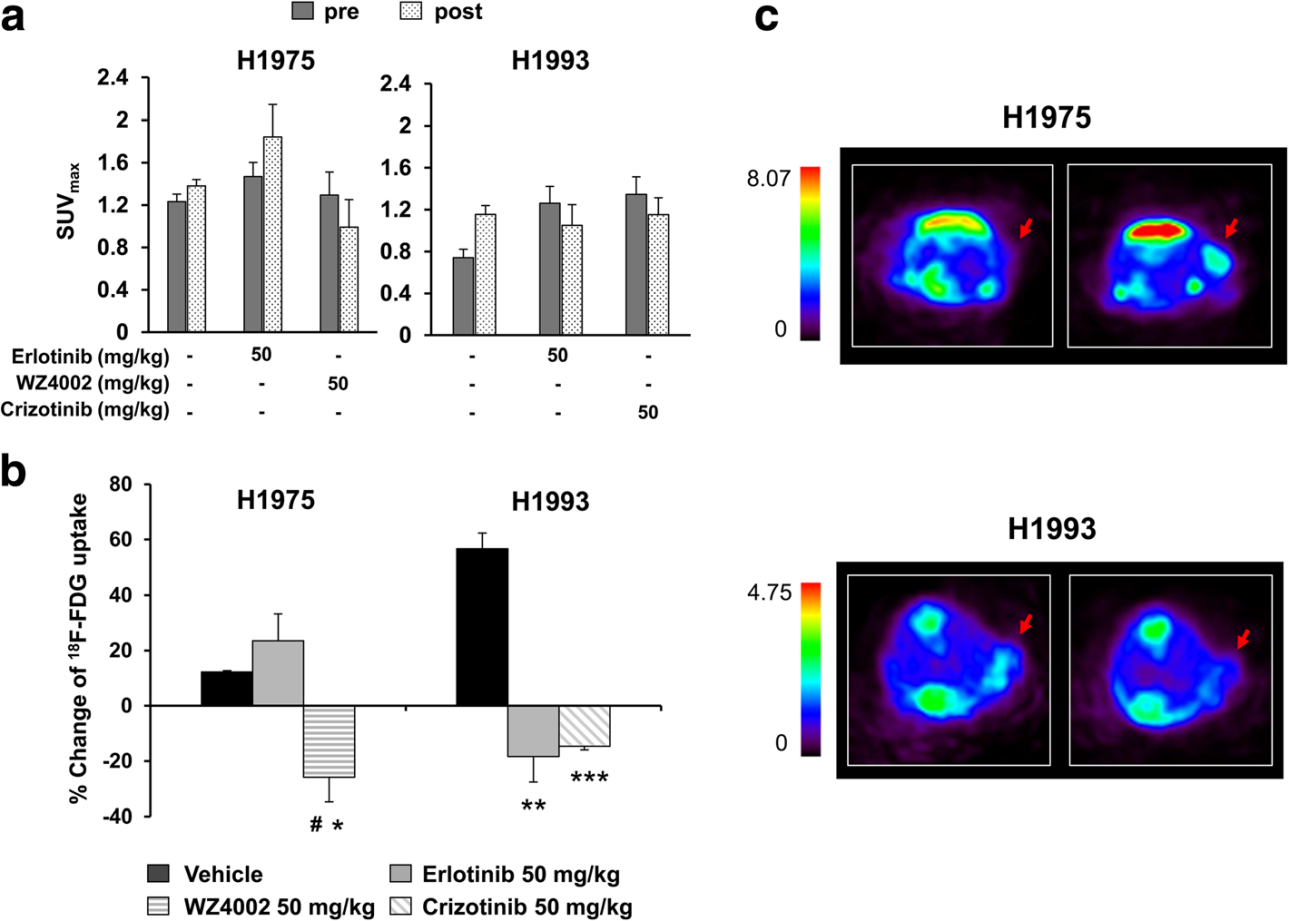
Imaging studies and percentage variation of ^18^F-FDG uptake in nude mice bearing H1975 and H1993 xenografts. **a** SUV_max_ of H1975 and H1993 xenografts obtained from baseline and post-treatment scans are expressed as mean ± SE. **b** Percentage variations of ^18^F-FDG uptake in H1975- and H1993-tumor-bearing animals in response to each treatment. Significant differences versus vehicle-treated animals were indicated with *p < 0.05, **p < 0.01, and ***p < 0.001; significant differences versus erlotinib-treated animals were indicated with ^#^ *p* < 0.05. **c** Representative transaxial ^18^F-FDG PET images of mice before (*left panels*) and 3 days after (*right panels*) treatment with 50 mg/kg erlotinib in H1975 (*upper panels*) and H1993 (*lower panels*) xenografts

Imaging studies of nude mice bearing H1975 and H1993 xenografts were performed with ^18^F-FDG PET/CT before and after 3 days treatment with different inhibitors or vehicle. All tumors were detected in the baseline ^18^F-FDG scan, and the SUV_max_ values obtained from pre- and post-treatment imaging studies are shown in Fig. 4a. The percentage variations of ^18^F-FDG uptake in post-treatment imaging studies compared with the corresponding baseline scans are shown Fig. 4b. In the H1975 xenografts, WZ4002 treatment caused a reduction of 25.87 % ± 8.93 % in ^18^F-FDG uptake (*n* = 4; *p* = 0.02) whereas the administration of erlotinib was followed by an increase of 23.51 % ± 9.72 %, a value not statistically different (*n* = 4; *p* = 0.49) from that found in vehicle-treated animals but significantly higher than that observed in WZ4002-treated mice (*p* = 0.01). In the H1993 xenografts, ^18^F-FDG uptake was reduced by 14.70 % ± 1.30 % (*n* = 4; *p* = 0.003) after treatment with crizotinib and by 18.40 % ± 9.19 % (*n* = 4; *p* = 0.13) in response to erlotinib. These values were significantly lower than that found in vehicle-treated animals (*p* = 0.006 vs erlotinib; *p* = 0.0006 vs crizotinib) indicating that both drugs reduce glucose consumption in H1993 tumors despite their resistance to erlotinib.

Representative PET transaxial images showing the differential metabolic response to erlotinib in H1975- and H1993-resistant xenografts are shown in Fig. 4c. As expected, the ^18^F-FDG uptake increased in H1975, tumor-bearing mice treated with erlotinib, whereas tracer uptake decreased in the H1993 xenografts after treatment with erlotinib. Therefore, the ^18^F-FDG uptake can be considered an early reliable biomarker of T790M-mediated resistance to erlotinib and sensitivity to third-generation EGFR TKIs. Conversely, in models of MET amplification, the ^18^F-FDG uptake does not reliably reflect this type of resistance since modulation of glucose consumption and proliferation appear to be uncoupled.

**Fig. 5:**
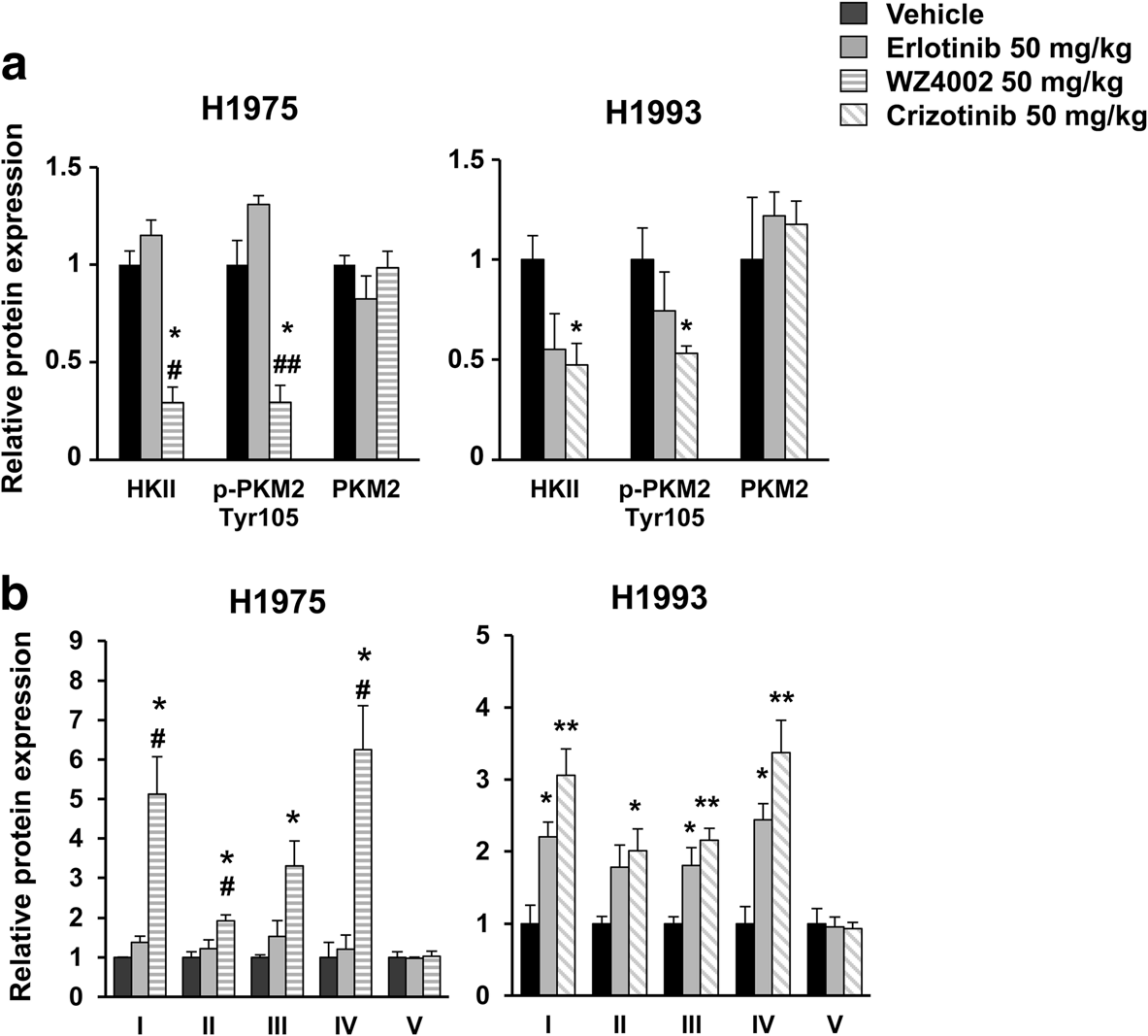
Tumor levels of glycolytic enzymes and OXPHOS in H1975 and H1993 xenografts. Surgically removed H1975 and H1993 tumors were homogenized and tested for levels of key glycolytic proteins (**a**) and subunits of mitochondrial complexes (**b**) by western blot analysis in response to each treatment. The bands were quantified by morphodensitometric analysis using ImageJ software. Data are expressed as relative protein levels of each treated sample compared to the corresponding vehicle-treated internal control. Significant differences versus vehicle-treated animals were indicated with *p < 0.05, **p < 0.01; significant differences versus erlotinib-treated animals were indicated with ^#^ *p* < 0.05 and ^##^ *p* < 0.01

After imaging studies, tumors were surgically removed in order to evaluate the expression levels of several key proteins of glycolysis and subunits of mitochondrial complexes (Fig. 5a, b). Figure 5a shows that a significant reduction of HKII and p-PKM2 Tyr105 occurs in H1975 tumors in response to WZ4002 as compared to untreated controls (*n* = 3; HKII, *p* = 0.03; p-PKM2 Tyr105, *p* = 0.04) or erlotinib-treated animals (*n* = 3; HKII, *p* = 0.02; p-PKM2 Tyr105, *p* = 0.0097). In the H1993 xenografts, treatment with crizotinib was followed by a significant reduction of HKII (*n* = 3; *p* = 0.03) and p-PKM2 Tyr105 (*n* = 3; *p* = 0.04) as compared to untreated controls and no statistically significant differences in the levels of the two glycolytic proteins were found between crizotinib- and erlotinib-treated animals (HKII, *p* = 0.73; p-PKM2 Tyr105, *p* = 0.34).

**Fig. 6:**
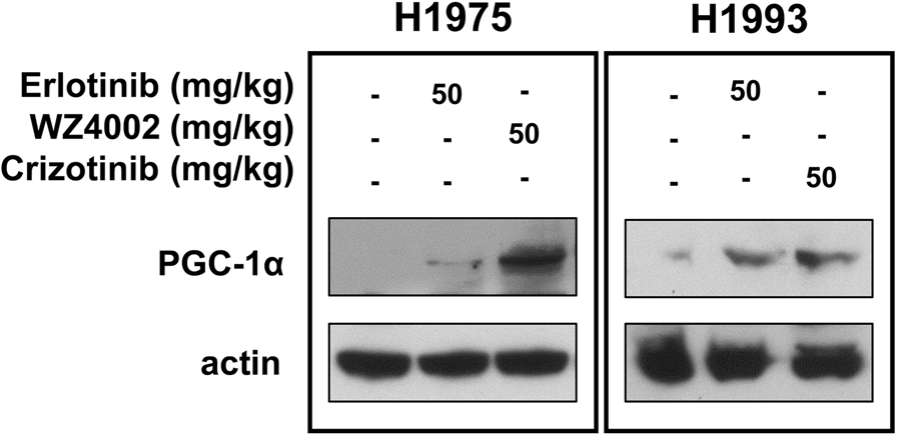
PGC-1α levels in xenografts obtained from untreated and treated animals. Levels of PGC-1α were determined in H1975 and H1993 excised tumors in response to the indicated treatment. Actin served to ensure equal loading

A parallel strong upregulation of mitochondrial complexes (subunits I–IV) was observed in H1975 tumors in response to WZ4002 as compared to vehicle- and erlotinib-treated animals (*n* = 3; *p* < 0.05 for all subunits) (Fig. 5b). Similarly in H1993 tumors, treatment with crizotinib caused a significant increase of OXPHOS levels (*n* = 4; *p* < 0.01 for subunits I, III, and IV; *p* < 0.05 for subunit II) as compared to vehicle-treated animals. Notably, in the same animals, even treatment with erlotinib caused a significant upregulation of mitochondrial complexes (*n* = 4; *p* < 0.05 for subunits I, III, and IV) as compared with vehicle-treated mice. In addition, levels of PGC-1α, a transcriptional coactivator of mitochondrial biogenesis, were increased after the treatment of H1975 and H1993 tumors with WZ4002 and crizotinib, respectively, whereas erlotinib was effective only in H1993 tumors (Fig. 6). These ex vivo findings taken together were in agreement with imaging results indicating that the reduction of aerobic glycolysis and the reactivation of oxidative phosphorylation underlie early ^18^F-FDG changes in response to EGFR inhibition.

## Discussion

The present study showed that ^18^F-FDG PET/CT is able to detect T790M-mediated resistance to EGFR inhibitors but it fails to identify MET amplification as a cause of resistance in NSCLC. An early reduction of ^18^F-FDG uptake was observed in H1975 tumors bearing the T790M mutation after treatment with WZ4002 but not with erlotinib. Conversely, in H1993 tumors, harboring MET amplification, ^18^F-FDG uptake was significantly reduced after treatment with both crizotinib and erlotinib despite the fact that they were resistant to erlotinib. In agreement with in vivo findings, a reduction of key glycolytic enzymes and an upregulation of OXPHOS were found in H1975 tumors only after treatment with WZ4002 whereas H1993 tumors showed a reduction of HKII and p-PKM2 Tyr105 as well as an increase of mitochondrial complexes after treatment with both crizotinib and erlotinib.

Previous studies with ^18^F-FDG PET/CT in erlotinib-naïve NSCLC patients showed that an early reduction of ^18^F-FDG uptake predicts morphovolumetric tumor response and correlates with longer PFS and OS [11, 12]. Our findings are in agreement with these observations by showing that effective inhibition of EGFR signaling pathways reduces ^18^F-FDG uptake as early as 3 days after initiation of therapy. Additional insights were also provided on the mechanisms modulating glucose metabolism in resistant tumors exposed to erlotinib showing that when resistance is due to redundant lateral signaling, the proliferation pathways remain constantly active whereas the glycolytic cascade is inhibited and accompanied by a PGC-1α-dependent upregulation of mitochondrial complexes.

In particular, the reduction of HKII levels in response to EGFR inhibition causes a decrease of glucose molecules entering the glycolytic cascade and a parallel reduction of ^18^F-FDG phosphorylation and tracer entrapment. PKM2 is another key glycolytic enzyme that catalyzes the conversion of phosphoenolpyruvate to pyruvate. High levels of PKM2 are found in a variety of human tumors including lung, breast, and colon cancer [25], and PKM2 is reported to exist as a low-activity dimeric or high-activity tetrameric form depending on the phosphorylation status of the enzyme at Tyr105 residue [26]. In the dynamic equilibrium between the two forms, the active tetrameric enzyme is favored by Tyr105 dephosphorylation. Therefore, it is conceivable that the reduction of p-PKM2 Tyr105 levels in response to EGFR inhibitors promotes tetramer formation and a high enzymatic catalytic activity in NSCLC that in turn may redirect pyruvate flux toward mitochondrial oxidative phosphorylation [27]. In agreement with these observations, OXPHOS levels were upregulated in animal models of T790M-mediated resistance after treatment with WZ4002 and in xenografts with MET amplification after therapy with erlotinib or crizotinib. Therefore, tumors that are resistant due to MET amplification are still able to switch energy metabolism from aerobic glycolysis to oxidative phosphorylation independently from the persistently high proliferation rate. As shown in our previous study in NSCLC cells, the metabolic switch from glycolysis to oxidative phosphorylation is not compensatory but the result of the fine tuning of the AKT and ERK1/2 pathways in NSCLC cells [16].

Our findings taken together may have many clinical implications for NSCLC patients receiving EGFR-targeted therapy and undergoing ^18^F-FDG PET/CT to monitor tumor response. In fact, the persistently high ^18^F-FDG uptake after therapy is a reliable imaging biomarker of tumor resistance whereas the early reduction of tracer uptake during treatment does not univocally indicate tumor sensitivity to EGFR inhibitors since glucose metabolism and proliferation may be differently regulated depending on the cellular context. Previous studies indicated that ^18^F-FDG uptake may identify early in the course of treatment NSCLC patients who may or may not benefit from treatment with EGFR TKIs [28]. This holds true for the majority of erlotinib-naïve NSCLC patients who have tumors with a low probability to be resistant. In erlotinib-treated patients, the possibility that the ^18^F-FDG uptake does not reflect sensitivity to EGFR TKIs should be taken into account especially when resistance is clinically suspected.

## Conclusions

In conclusion, ^18^F-FDG uptake is a reliable imaging biomarker of T790M-mediated resistance and its reversal in NSCLC whereas it may not be accurate in the detection of MET-mediated resistance. When resistance to EGFR TKIs is due to redundant lateral signaling, the proliferation pathways remain constantly active while an early metabolic switch from aerobic glycolysis to oxidative phosphorylation may occur in resistant tumors reducing their dependence from external glucose supply and tracer uptake.

## Abbreviations

^18^F-FDG: ^18^F-Fluorodeoxyglucose
CT: Computed tomography
EGFR: Epidermal growth factor receptor
HKII: Hexokinase II
NSCLC: Non-small cell lung cancer
OS: Overall survival
OXPHOS: Mitochondrial complexes for oxidative phosphorylation
PET: Positron emission tomography
PFS: Progression-free survival
PGC-1α: Peroxisome proliferator-activated receptor-γ coactivator-1α
PKM2: Pyruvate kinase M2
TKIs: Tyrosine kinase inhibitors
